# Decolonial perspectives on climate change: Learning from the Kainai First Nation in Canada

**DOI:** 10.1177/26349825251323144

**Published:** 2025-04-17

**Authors:** Ranjan Datta, William Singer-III, Jebunnessa Chapola

**Affiliations:** Mount Royal University, Canada; Kainai First Nation Elder and Land-Based Educator, Canada; University of Regina, Canada

**Keywords:** Climate change, decolonial learning, Indigenous knowledge, traditional land-based knowledge, Indigenous Elders, adaptation, resilience, sustainability

## Abstract

This study focuses on the reflections and insights of Indigenous Elders from the Kainai First Nation in Canada regarding climate change challenges and potential solutions. Through a decolonial and Elder-led land-based learning process, the research team captured the traditional land-based knowledge of the Elders, rooted in their profound understanding of the interconnectedness between humans, nature, and climate. The findings showcase the shared concerns of Indigenous Elders and emphasize the imperative of recognizing and valuing Indigenous knowledge systems as crucial resources for climate adaptation and mitigation strategies. Indigenous land-based knowledge offers a holistic perspective that encompasses social, cultural, and spiritual dimensions, advocating for sustainable practices and harmonious coexistence with the environment. This decolonial study identifies specific strategies and practices proposed by Indigenous Elders as potential solutions to climate change challenges. The insights shared by Indigenous Elders emphasize the urgency of integrating Indigenous knowledge systems into global efforts to address climate change. By honoring and learning from their wisdom, societies can cultivate a more holistic and sustainable approach to climate adaptation and mitigation, fostering resilience, biodiversity conservation, and the well-being of both human and non-human communities.

## Introduction

The intensifying impacts of climate change—such as rising temperatures, prolonged heatwaves, unpredictable weather patterns, and extreme events like wildfires and floods—are felt acutely across the globe, with Western Canada particularly vulnerable. These shifts pose significant challenges to traditional ways of life, particularly for Indigenous communities, who experience these changes more profoundly due to their deep-rooted connection to the land (McKinley, 2023; Nuttall, 2016; Treat et al., 2021). Indigenous peoples in Western Canada are at heightened risk from the climate crisis due to their deep interconnection with the land, which is foundational to their cultural, spiritual, and physical well-being.

As Indigenous scholars like McGregor (2018, 2020) and Coulthard (2014) highlight, the climate crisis exacerbates historical and ongoing colonial impacts, further marginalizing Indigenous communities by threatening traditional practices, food security, and territorial sovereignty. The rapid environmental changes disrupt ecosystems that Indigenous peoples have relied upon for millennia, making it increasingly difficult to sustain traditional livelihoods and cultural practices. As Indigenous scholar Whyte (2018) argues, the climate crisis intersects with colonialism to create a “double exposure” where Indigenous communities face both environmental degradation and socio-political disenfranchisement. This confluence of factors places Indigenous peoples in Canada at a disproportionate risk, requiring urgent attention and the inclusion of Indigenous knowledge in climate adaptation strategies. Indigenous perspectives on climate change are critically underrepresented in research, policy, and decision-making processes. This gap not only overlooks the valuable knowledge held by Indigenous peoples—knowledge that is grounded in centuries of lived experience and environmental stewardship—but also exacerbates the vulnerabilities these communities face (Castleden and Skinner, 2014; Lavallee and Poole, 2010; Whyte, 2018). Environmental changes disrupt Indigenous traditional practices, spiritual connections, and overall health, leading to a range of physical and mental health challenges, from increased rates of diseases like diabetes and cancer to heightened instances of depression caused by disconnection from the land (Castleden and Skinner, 2014; Ford, 2008; Furgal and Seguin, 2006; Lavallee and Poole, 2010; Palinkas and Wong, 2020).

Addressing these research gaps from and within Indigenous community perspectives is critical. Indigenous land-based approaches offer holistic, ecologically attuned approaches to climate adaptation and resilience (Datta, 2020, 2023; Datta et al., 2024). Indigenous land-based approaches to dealing with the climate crisis emphasize the restoration and stewardship of natural ecosystems, drawing on traditional ecological knowledge that fosters sustainable relationships with the environment (Kimmerer, 2013; Simpson, 2017). These practices prioritize community-led conservation efforts, seasonal knowledge, and the revitalization of Indigenous governance systems to build resilience against climate impacts. By creating meaningful integration between Indigenous land-based knowledge into climate policies and practices, we can move toward more sustainable decision-making frameworks that respect Indigenous environmental sustainability and support decolonial practices (Skinner et al., 2014; Whyte, 2018). Therefore, decoloniality involves not only acknowledging and addressing historical injustices but also recognizing and valuing existing Indigenous knowledge systems (Holtgren, 2013; McCracken, 2019; Norman et al., 2020).

This article emerges from our collaboration with an Indigenous Elder William Singer III from the Kainai First Nation at Treaty 7 Territory in Western Canada, who has generously shared their wisdom on land-based knowledge and practices. As Indigenous Elder William, racialized immigrants, and feminist scholars, we tried to decolonize the meanings of the climate change crisis and center Indigenous land-based solutions. Through Indigenous Elder’s teachings, we aim to deepen our understanding of land-based knowledge and integrate these insights into our research and educational practices.

Our approach begins with a reflection on our positionality, followed by an exploration of the Elder’s community experiences and reactions to climate change. We then address the challenges and future directions identified by the Elder, concluding with our reflections on the most critical lessons learned throughout this process.

## Positionality

We see it as our responsibility to discuss and present our positionality. This is a significant aspect of research on Indigenous and racialized communities. We acknowledge and respect that we live and work on the traditional territories of the homelands of the Niitsitapi (the Siksika, Kainai, and Piikani nations), the Îyârhe Nakoda, and the Tsuut’ina. We are grateful for the Elder, who helped us to learn from his knowledge and experiences. The following sections present who we are as relational researchers, where we are coming from, why we are doing this research, and our transformation from our learning.

Author 1: I am a settler of color who has lived and worked on Treaty 6 and 7 territories for the past 13 years. As a racialized scholar, I have spent the last 17 years developing and applying decolonial and anti-racist research frameworks in collaboration with Indigenous and non-Indigenous communities across Canada and South Asia. My research is grounded in a lifelong commitment to relational accountability and is supported by a robust network of Indigenous, visible minority immigrant and refugee, and Black communities, scholars, and practitioners. For me, research is a sacred responsibility—a practice that connects people and knowledge across cultures and generations.

Author 2: Elder William Singer III, a co-researcher and land-based educator from Kainai First Nation, is an environmental advocate and climate change expert. Rooted in Blackfoot culture and worldview, Elder Singer uses his artistry to teach and inspire others, combining traditional knowledge with modern activism. He is also an entrepreneur, an organizer of spiritual and cultural events, and a steadfast advocate for First Nations rights and knowledge. His teachings remind us of the power of art and land-based learning in addressing today’s challenges.

Author 3: I, too, am a settler and a woman of color trained as a decolonial, anti-racist, feminist activist scholar. Over the past decade, I have been deeply engaged in community-building and social justice activism alongside Indigenous communities. My work is informed by a commitment to collaborative learning and transformative change.

Together, our diverse perspectives enrich our shared commitment to decolonial research and advocacy. Through these relationships, I am constantly reminded that knowledge is not only discovered but also lived, shared, and nurtured collectively.

## Theoretical framework and methods

We used the decolonial theoretical framework in our research as it is a vital approach to environmental sustainability that centers on the Indigenous community needs, revitalization, and self-determination of Indigenous peoples and their knowledge systems (Kovach, 2009, 2015; Smith, 2013; Wilson, 2008). This framework emerged in response to the enduring impacts of colonial legacies and is committed to decolonizing research practices, challenging Western environmental policies and practices that have contributed to numerous ecological crises, and affirming Indigenous sovereignty and self-determination (Kovach, 2009; Smith, 2013; Wilson, 2008). The decolonial framework prioritizes Indigenous worldviews, epistemologies, and ways of knowing, recognizing Indigenous land-based learning and practice as scientific and valuable forms of knowledge historically marginalized by colonial powers (Absolon, 2011; Datta, 2024). By restoring and reclaiming sustainable Indigenous land-based knowledge within environmental research contexts, this approach promotes the active participation and leadership of Indigenous communities throughout the research process (Chilisa, 2012). It places Indigenous voices, perspectives, and priorities at the center of research, striving to address Indigenous communities’ specific needs and aspirations. The decolonial framework supports Indigenous peoples’ well-being, resilience, and self-determination by reclaiming and revitalizing Indigenous knowledge systems and effectively challenging entrenched power imbalances (Smith, 2013).

**Figure 1.**
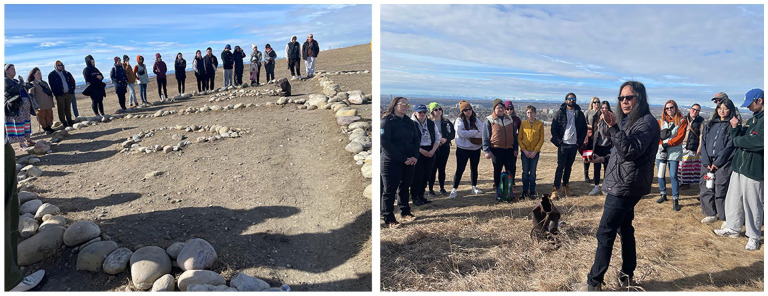
The image on the right illustrates how Indigenous and non-Indigenous community members are learning from an Elder’s Medicine Wheel teachings, while the second photo captures Kainai First Nation Elder William Singer III imparting land-based learning knowledge.

**Figure 2.**
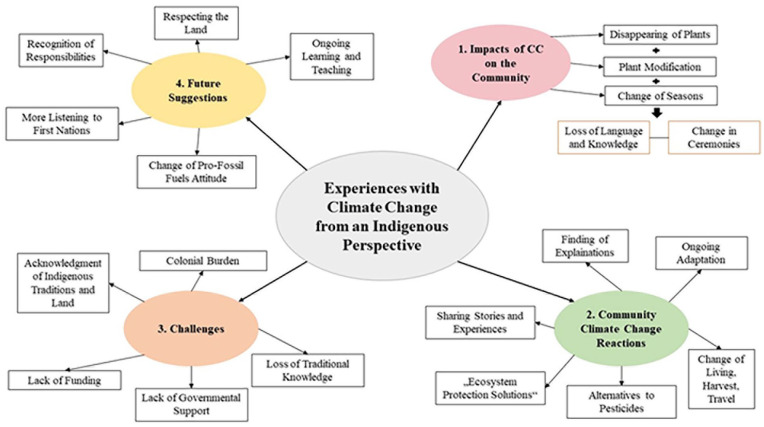
Indigenous perspectives on climate change impacts, challenges, and land-based adaptions within and from the community.

Following the decolonial research framework, we use deep listening from Indigenous Elder William Singer III. Deep listening in Indigenous research is a critical process of active, respectful, and culturally attuned engagement with Indigenous voices, perspectives, and knowledge systems. It involves more than simply hearing words from Elder William; it requires researchers to be respectful and implement stories in their practice that they are listening to, acknowledging the significance of oral traditions and non-verbal communication (Kovach, 2009; Smith, 1999). Through deep listening, we honored the complexity and richness of Indigenous knowledge, recognizing it as an essential science in understanding the unique experiences, challenges, and aspirations of Indigenous peoples. Our approach was rooted in the principles of reciprocity and relational accountability, ensuring that the research process was collaborative and mutually beneficial (Wilson, 2008). By prioritizing deep listening, we aimed to co-create knowledge that strongly supports Indigenous self-determination, resilience, and well-being, rather than imposing external frameworks or interpretations (Absolon, 2011). This method also emphasizes the importance of trust-building and long-term relationships, which are crucial for meaningful and respectful engagement with Indigenous communities (Chilisa, 2012). Therefore, deep listening in this research was not just a methodological choice but a commitment to responsible research practices that center and affirm Indigenous sovereignty and knowledge systems (Kovach, 2009). Figure 1 and 2 showcases, our respectful deep listening with Kainai First Nation Elders in 2022, 2023, and 2024.

In addition to deep listening, we also learned from Kainai Elder-led (i.e., Elder William) land walks for our learning from 2021 to 2024 every summer. Indigenous Elder-led land walks are critical in Indigenous research because they embody the transmission of knowledge, culture, and spirituality directly connected to the land. These walks allow Elder to share their deep understanding of the land’s history, its ecosystems, and the stories embedded within it, providing a living context for Indigenous knowledge that is often inaccessible through conventional research methods (Simpson, 2017). By walking the land with Elder, researchers can engage in experiential learning, where knowledge is not just shared but felt and lived, fostering a deeper connection to Indigenous worldviews and epistemologies (Cajete, 1999). This method also reinforces the principle of relationality, emphasizing the importance of relationships between people, land, and all living beings, which is central to Indigenous ways of knowing (Wilson, 2008). Moreover, Elder-led land walks honor the knowledge of Elders, ensuring that research remains grounded in community priorities, cultural practices, and the preservation of traditional knowledge (Tuck and McKenzie, 2015). Such as Figure 1 illustrates a Kainai Elder William Singer III conveying the profound significance of the land to cross-cultural youths through immersive land-based walks intertwined with engaging storytelling sessions. During our landwalk, we used individual journals to reflect on our learning and take notes. The Elder William leading the walk also contributed as an author to guide this article. Throughout this document, we adhere to their guidelines and respectfully request consent for inclusion.

In Figure 1, both images show how Kainai Elder William Singer III (second author) has been explaining the significance of the land to cross-cultural youths through a combination of land-based walks and storytelling sessions.

## Finding analysis

We listened to the recorded stories several times and categorized the findings into themes and subthemes. Deep listening during story-sharing and re-listening from the recordings helped us to understand the interconnections and meanings. Furthermore, it also allowed us (non-Indigenous racialized land-based researchers such as authors 1 and 3) to reflect and request our learnings individually. Storytelling from Indigenous Elder is often combined with personal experiences and stories and learnings from past generations. Often it happens that they share stories that they learned from their grandparents. Especially these moments show the long-lasting roots and generational knowledge in Indigenous communities.

In our analysis, we prioritize Elder William’s guidelines and perspectives and topics that are essential from his point of view. We as non-Indigenous racialized land-based researchers (such as authors 1 and 3) see ourselves as listeners and learners; therefore, our analysis represents firsthand traditional knowledge. Before publishing our work, we reconnect again with Elder William (i.e., second author) and present our findings to them. In doing so, we ensure that the results and their knowledge are presented correctly. Beyond that, we want them to be part of our work and to guide us (non-Indigenous racialized land-based researchers such as authors 1 and 3) in every step.

## Findings: Decolonial learning reflections

The deep listening and land walk with the Elder were very critical. We created categories to structure his shared land-based knowledge, thoughts, and lived experiences. The presentation of the results starts with describing the impacts of climate change on the Kainai Elder William Singer III (i.e., second author) and his community. Furthermore, the reactions and responses of his community to climate change are presented. Then, the challenges faced by his community are analyzed, and finally, he has made many suggestions for future deliberations and changes that will be discussed at the end. Figure 1 provides a good overview and summary of the findings.

Figure 1: Overview of the findings categorized into main and subthemes. This outlines the experiences of the Kainai First Nation Elders with climate change from his perspective, divided into four key areas: **(1) impacts of climate change** on the community include the disappearing of plants, changes in seasons, and alterations in ceremonies, leading to a loss of language and knowledge; **(2) community reactions** involve finding explanations, adapting to changes, and exploring alternatives to pesticides; **(3) challenges** faced by Indigenous communities include a lack of funding, governmental support, and the loss of traditional knowledge; and finally, **(4) future suggestions** emphasize the importance of respecting the land, ongoing learning and teaching, and the need for a shift in attitudes toward fossil fuels, alongside increased recognition and listening to Indigenous voices.

## Impacts of climate change on the community

The impact of climate change varies in every Indigenous community differently. The Elder explained changes in his Kainai First Nation community and how these affect their culture and traditions. One significant challenge that we learned about was the Native plant disappearance and/or modification of plants. As Elder explained the Elders’ aunt used to go out into nature and harvest herbs and specific plants for cooking, healing, and ceremonies. However, these traditional plants are nowadays harder to find or impossible to harvest because of more prolonged and more extensive roots. As the Kainai Elder William Singer III (Second author) explains: *“This is because of climate change.”* Furthermore, the seasons are changing, and he adds, *“You can’t work with the seasons anymore.”* These significant changes impact Indigenous communities and their traditions immensely. With the disappearance of the plants, the knowledge and the associated language are also lost. Beyond that, ceremonies must resort to alternatives that change the content and the connection. The Elder William mentioned *that “our communities lost much land-based information due to climate change. We lost a lot of our land-based language and ceremonies.”* Much knowledge is already gone and not recoverable for future generations. As Elder William explained support from the government is often lacking in Indigenous communities. Therefore, Indigenous communities create solutions and reactions to climate change and environmental problems themselves. For years, they have been working on how to explain climate change. The changing environment is hard to understand, especially for community elders. The Elder William describes climate change as: “*The more grazing you put on the land, the more you compact it. And the more you compact the land, the roots in that particular soil will either die or you work your way deep.”* William explains the effect of overgrazing and overuse of land, which leads in the long term to degradation and infertile soils. He also talks about the impact of climate change with the help of stories. He told us the story about Nappi’s playground. In this story, Nappi loses all the animals, plants, and environment in a gambling game. Everything was kept behind a vast dam. Nappi was able to win all of it back. Therefore, he had to destroy the dam, which caused a considerable flood. Finally, everything started to fall back into place again. But the Elder William described, *“Things are not the same anymore. All the changes that Nappi created still affect us to this day. Some of them are negative, which was him playing around with the environment. He had the power to do that.”* As William adds, the overall message of this story and the connection to climate change is, *“We are responsible for these changes.”* Humans destroy the environment, but they also have the power to stop this destruction if they want to. We can make these decisions, but it is questionable if we can make the right ones.

Adaptation to climate change is mandatory for survival. The Elder William reports that *“climate change is affecting everybody”*; therefore, everybody needs to act and adapt to it. Furthermore, he explains that *“we have been adapting to situations ever since colonization . . . We have been adapting to what has been presented to us. We don’t have a choice in that.”* The history of colonization brutally shaped Indigenous people land-based culture and practice, but they survived and adapted. Indigenous people can adapt, but that does not mean they should constantly adapt to everything Western societies dictate. Nevertheless, Elder William reports that Indigenous people are changing their living, harvest, and travel habits. They try to reduce their impact on the environment and, therefore, protect their surroundings. They would also like to implement more changes, such as energy production with solar and wind. However, these adaptation methods lack capital and government funding and support. The Elder William community is doing everything they can, and he developed a plan for ecosystem protection solutions. With this plan, he tried to mitigate climate change factors, such as stopping fossil fuel production on the reserve. He has been trying to find alternatives to the use of pesticides. William explained to cross-cultural communities how climate change mitigation and adaptation are possible without colossal capital, even if it is more complex. Beyond that, he emphasized the importance of storytelling and sharing experiences. Communication with each other is one of the key elements to improve situations and build resilience.

## Challenges in climate crisis

Indigenous communities are facing many challenges connected to climate change and historical and ongoing environmental colonization. Colonialism and residential schools caused Indigenous to disconnect from their land-based culture and traditions. Without addressing historical injustice, restoring climate adaptation is difficult and is affected by the current changes. Thus, Indigenous Elders’ land-based knowledge and practice are critical for dealing with the climate crisis.

Lack of enough governmental support is a critical challenge in dealing with the climate crisis. For instance, Elder William reported that the government is giving them *“just the bare minimum.”* They lack support and funding for clean water, infrastructure, roads, and education. Furthermore, he responds, *“They will never give you the money, even if I am applying for it. They won’t fund me. Even for the climate change department. We never fully get what we deserve.”* Ongoing mistreatment and exclusion are still on the agenda; getting heard and listened to is one of the significant challenges. Therefore, for the official recognition of Indigenous traditions and lands, it is necessary to have more communication and cooperation. The Elder William emphasizes that they are very willing for more relationship building and collaborations. His community is seeing the necessity and importance of it. He adds, *“What happens affects us. We are all involved. Our goal is to restore a lot of these places on our reserve. We are working with everyone, even the government.”* Furthermore, he explains that *“we are not selfish people even after all we went through. Our elders always tell us to make friendships.”* Indigenous communities are open to communication and working together. They are willing to share their land-based knowledge and find solutions that benefit all. The only thing lacking is motivation and action from the governmental side.

## Land-based adaptions

As Elder William explained, “how severe the future impact of climate change will be is still unknown. Humans have the chance to keep the changes as small as possible.” During the story-sharing with the Elder, he pointed out land-based adaption on how to proceed in the future. Indigenous land rights are crucial for building meaningful relationships between the government and Indigenous Nations to deal with the climate crisis. Therefore, climate policymakers need to start listening to and respecting Indigenous land-based knowledge and practice. Elder also suggested strong Indigenous land rights advocacy as a significant part of climate crisis adaptation as Indigenous people know how to protect their land and environment.

The Elder William formulates the message regarding the government “*You need to listen to First Nations . . . they are listening to many of the surface things, but there is much more than that.”* Meaningful collaboration and possibilities for communication are highly recommended to support further. Talking needs to be turned into action. Indigenous communities already have many adaptation and mitigation solutions, and sharing experiences, research, and outcomes benefits both sides. A considerable challenge, thereby, is the mind-set change of the government. The Elder William reports that “*the government here is pro-fossil fuels, so they won’t support us because we are not moving in that direction. And that’s a shame. If you are destroying the land, you get money for it.”* The support of fossil fuels is one of the key drivers of climate change, and a community should get more funding and support if they are trying to eliminate this economic driver than they would earn by supporting it.

Respecting the land and the environment is essential to minimize climate change’s impact. As Elder William suggested everyone has the responsibility to protect the environment. Everyone should take responsibility to protect their water for their future generation. The Elder William reminds us, *“Living in this world, we must respect it, but we don’t own it.”* We are all guests on this earth, and we should act like guests: respectful and mindful. Therefore, we need ongoing land-based learning and teaching. The Elder emphasizes that *“the importance is to teach our community . . . [and] we have the control over it . . . our reserve can be an example for other nations.”*

Thus Indigenous land-based learning is crucial for addressing the climate crisis because it integrates traditional Elders’ land-based knowledge with sustainable practices, reclaiming a deep connection to the land. This approach emphasizes respect for traditional knowledge, promoting conservation and biodiversity. By learning from Indigenous knowledge, we can adopt more holistic and long-term strategies for environmental stewardship. It also challenges Western-centric models of development that often contribute to environmental degradation. It advocates for Indigenous communities’ needs, ensuring their voices and practices are central to global climate solutions.

## Discussions

In the contemporary discourse of Western research and policymaking, has emerged as significant challenges, particularly regarding the climate crisis (Smith, 2013; Tuck and Yang, 2012). Decolonial perspectives push for a fundamental re-evaluation of history, one that begins with educational curricula designed to foster critical thinking about Indigenous communities and their past (Battiste, 2013; McCracken, 2019). This approach is more than a quest for social justice; it challenges entrenched systems of racism, capitalism, and heterosexism ((Barker, 2017; Razack, 2015), calling for a paradigm shift that includes Indigenous perspectives (Coulthard, 2014; Davis et al., 2016). Such a shift is especially crucial in addressing global issues like climate change, where Indigenous knowledge, particularly that of the Kainai First Nation in Canada, offers invaluable insights into sustainable practices and environmental stewardship.

Elder-led Climate Action Learning. Decoloniality, as practiced by the Kainai First Nation, is not just a theoretical concept, but it is an active, lived experience rooted in the land. The Kainai people, part of the Blackfoot Confederacy, have long maintained a deep connection to their traditional territories, drawing on a wealth of land-based knowledge passed down through generations. This knowledge is not only central to their cultural identity but also to their approach to climate action (Battiste and Henderson, 2000; McGregor, 2018; Simpson, 2017; Wilkinson, 2008). Elder-led Land-based learning, a practice where education is directly tied to the land and its ecosystems, embodies this decolonial approach (Chilisa, 2012; Snelgrove et al., 2014). It serves as both a form of resistance to colonial structures and a proactive strategy for climate change mitigation and adaptation (Adelson, 2005; Leddy, 2017). In Western societies, economic and political systems are often dominated by extractive practices that prioritize short-term gains over long-term sustainability (Ladson-Billings, 2021; Vosko, 2022). This Western approach has led to environmental degradation on a global scale, exacerbating the climate crisis (Davis et al., 2016; Kind and Pasternak, 2018). In contrast, we also learned from our study that Elder-led land-based learning emphasizes a reciprocal relationship with the environment, where human actions are guided by respect for the land and a commitment to its health and vitality. This perspective challenges the dominant Western narrative that views land primarily as a resource to be exploited (McGregor, 2018; Simpson, 2017).

The experiences and narratives shared by Kainai Elders are particularly instructive in this regard (Alfred, 2014; Battiste, 2013; Castellano, 2004; Greenwood, 2008; Wildcat, 2013). Their teachings emphasize the importance of resilience, hope, and the power of forgiveness, even in the face of profound adversity (Castellano, 2004; Chilisa, 2012). These values are not only relevant to their own community but also offer lessons for the broader society as it grapples with the challenges of climate change (McCracken, 2019; Smith, 2013). We also learned from our study that the Kainai First Nation’s land-based learning initiatives serve as a model for how Indigenous knowledge can be harnessed to foster environmental stewardship and address the climate crisis.

Decolonial Land-based Climate Action. For meaningful climate action, Western policymakers must integrate these decolonial perspectives, recognizing the value of Indigenous knowledge systems (Coulthard, 2014; McCracken, 2019). This decolonization requires a paradigm shift that moves away from the anthropocentric and capitalistic models currently prevailing in global climate strategies (Tuck and Yang, 2012). Instead, it advocates for a holistic approach that sees humans as part of a broader ecological system, one that must be maintained and respected for the survival of all species, including our own (Battiste, 2013; Snelgrove et al., 2014).

Land-based practices are closely tied to decoloniality and involve establishing trust and respect between Indigenous and non-Indigenous communities (Alfred, 2014; Razack, 2015). It is a long-term process that demands a critical reassessment of cultural narratives and belief systems that have historically marginalized Indigenous voices ((Battiste and Henderson, 2000; McGregor, 2018). We also learned from our study that land-based learning and practice as climate action is demonstrated through their openness to collaboration and dialogue with the broader society. Their land-based learning initiatives are not only a means of preserving their cultural heritage but also a way to share their environmental wisdom with the world. This sharing is crucial in the context of climate change. The Kainai First Nation, like many Indigenous communities, is on the front lines of the climate crisis, facing immediate and severe impacts on their land and way of life. At the same time, they possess knowledge and practices that can inform broader climate strategies (Davis et al., 2016). For instance, their understanding of local ecosystems, seasonal changes, and sustainable resource management offers practical solutions that are often overlooked in Western scientific approaches (Alfred, 2014; Simpson, 2017).

However, the benefits of decolonial land-based knowledge can only be fully realized if there is a genuine commitment to climate action (DiAngelo, 2018; Kind and Pasternak, 2018). This means not only acknowledging the historical injustices faced by Indigenous peoples but also actively working to dismantle the systems of racism and oppression that continue to marginalize them (McCracken, 2019; Razack, 2015; Snelgrove et al., 2014; Wilkinson, 2008). In Canada, where the rhetoric of decolonization is often at odds with the reality of ongoing colonial practices, there is a pressing need for policymakers to bridge this gap (Greenwood, 2008; Leddy, 2017; Miller, 2007; Vosko, 2022). This includes ensuring that Indigenous voices are not only heard but are central to decision-making processes, particularly in areas such as environmental policy and climate action (Coulthard, 2014; McGregor, 2018).

Realizing a decolonial and reconciled society is a complex and ongoing process (Adelson, 2005; Castellano, 2004; DiAngelo, 2018; Kind and Pasternak, 2018). It requires a collective effort to dismantle deeply entrenched systems of racism and misrepresentation that continue to oppress Indigenous communities (Leddy, 2017; Snelgrove et al., 2014). This is not just the responsibility of policymakers but of every individual who benefits from the current systems (Coulthard, 2014; Wilson, 2019). Respecting Indigenous peoples and their environment is not only a moral imperative but also a practical one if we are to effectively address the global climate crisis (McCracken, 2019; Vosko, 2022).

## Conclusion

In conclusion, the insights provided by the Kainai First Nation through their land-based learning initiatives highlight the transformative role of Indigenous knowledge systems in addressing the climate crisis. These initiatives challenge colonial paradigms by offering relational and reciprocal frameworks that prioritize sustainability and respect for the natural world (Battiste and Henderson, 2000; McGregor, 2018). As relational researchers, we recognize our positionality in engaging with this work. Author 1 brings over 17 years of experience in decolonial and anti-racist research with Indigenous and racialized communities, guided by a commitment to relational accountability. Author 2, Elder William Singer III, embodies Blackfoot worldviews and teachings through his work as an environmental educator, artist, and advocate, offering invaluable perspectives rooted in traditional knowledge. Author 3, a woman of color and activist scholar, draws from her experiences in feminist, anti-racist, and decolonial community-building with Indigenous communities.

Acknowledging our diverse positionalities allows us to approach this research with humility, reciprocity, and respect, ensuring that Indigenous voices remain central to climate strategies. Integrating Indigenous perspectives into broader climate frameworks requires a commitment to decoloniality and active confrontation of systemic inequities that have historically marginalized these knowledge systems (Simpson, 2017). This work extends beyond decolonization to emphasize the leadership and sovereignty of Indigenous peoples in shaping pathways toward climate justice. By embracing these teachings, we collectively contribute to building equitable, sustainable, and culturally rooted responses to the pressing environmental challenges of our time (Alfred, 2014; Smith, 2013).
